# Supplementary material for: Snotwatch COVID-toes: An ecological study of chilblains and COVID-19 diagnoses in Victoria, Australia
Source: PLOS Glob Public Health. 2022 Oct 19;2(10):e0000488. doi: 10.1371/journal.pgph.0000488 (PMC10022016; doi:10.1371/journal.pgph.0000488)
Supplement: S1 File — Table A: COVID-19, suspected COVID-19 and temperature associations with chilblains with leads and lags. (DOCX) [file pgph.0000488.s001.DOCX]

# S1 File

# Appendix A: Chilblains, COVID-19 disorder and Suspected COVID-19 disorder lead and lag analysis results

Table A: COVID-19, Suspected COVID-19 and Temperature associations with Chilblains with leads and lags

|  | COVID-19 Disorder risk Ratio (99%CI) | Suspected COVID-19 Risk Ratio (99%CI) | Decreasing Temperature (per 1°C) Risk Ratio (99%CI) |
| --- | --- | --- | --- |
| Chilblains Lag 8 | 1.05 | 6.08 | 1.32 |
| Chilblains lag 7 | 0.70 | 8.14 | 1.32 |
| Chilblains lag 6 | 1.04 | 6.76 | 1.31 |
| Chilblains lag 5 | 1.34 | 5.57 | 1.31 |
| Chilblains lag 4 | 1.66 | 4.90 | 1.32 |
| Chilblains lag 3 | 2.69 | 3.51 | 1.31 |
| Chilblains lag 2 | 1.96 | 4.45 | 1.30 |
| Chilblains lag 1 | 1.73 | 5.03 | 1.30 |
| No lead or lag | **5.72** | **3.23** | **1.33** |
| Chilblains Lead 1 | 1.57 | 5.71 | 1.30 |
| Chilblains Lead 2 | 2.24 | 4.15 | 1.31 |
| Chilblains Lead 3 | 2.52 | 3.68 | 1.30 |
| Chilblains Lead 4 | 2.27 | 4.49 | 1.30 |
| Chilblains Lead 5 | 1.72 | 5.01 | 1.31 |
| Chilblains Lead 6 | 1.65 | 4.87 | 1.31 |
| Chilblains Lead 7 | 1.74 | 4.97 | 1.32 |
| Chilblains Lead 8 | 4.06 | 2.93 | 1.32 |
