# Supplementary material for: Snotwatch COVID-toes: An ecological study of chilblains and COVID-19 diagnoses in Victoria, Australia
Source: PLOS Glob Public Health. 2022 Oct 19;2(10):e0000488. doi: 10.1371/journal.pgph.0000488 (PMC10022016; doi:10.1371/journal.pgph.0000488)
Supplement: Attachment — Submitted filename: Response to Reviewers.docx [file pgph.0000488.s002.docx]

Response to reviewers.
Journal Requirements:

1. In ethics statement in the manuscript and in the online submission form, please provide additional information about the patient records/samples used in your retrospective study. Specifically, please ensure that you have discussed whether all data/samples were fully anonymized before you accessed them and/or whether the IRB or ethics committee waived the requirement for informed consent. If patients provided informed written consent to have data/samples from their medical records used in research, please include this information.

- A statement about the data has been included in the Ethics Approval section. Line 132.

2. Please amend your detailed Financial Disclosure statement. This is published with the article. It must therefore be completed in full sentences and contain the exact wording you wish to be published.

a. Please clarify all sources of funding (financial or material support) for your study. List the grants (with grant number) or organizations (with url) that supported your study, including funding received from your institution. 
b. State the initials, alongside each funding source, of each author to receive each grant.
c. State what role the funders took in the study. If the funders had no role in your study, please state: “The funders had no role in study design, data collection and analysis, decision to publish, or preparation of the manuscript.”
d. If any authors received a salary from any of your funders, please state which authors and which funders.

- The funding disclosure statement has been included following the bibliography. Lines 404-407

3. Please ensure that your Financial disclosure statement is matched with the funding information.

- As above.

4. Figure 1.png: please (a) provide a direct link to the base layer of the map (i.e., the country or region border shape) and ensure this is also included in the figure legend; and (b) provide a link to the terms of use / license information for the base layer image or shapefile. We cannot publish proprietary or copyrighted maps (e.g. Google Maps, Mapquest) and the terms of use for your map base layer must be compatible with our CC-BY 4.0 license.

Note: if you created the map in a software program like R or ArcGIS, please locate and indicate the source of the basemap shapefile onto which data has been plotted.

If your map was obtained from a copyrighted source please amend the figure so that the base map used is from an openly available source. Alternatively, please provide explicit written permission from the copyright holder granting you the right to publish the material under our CC-BY 4.0 license.

Please note that the following CC BY licenses are compatible with PLOS license: CC BY 4.0, CC BY 2.0 and CC BY 3.0, meanwhile such licenses as CC BY-ND 3.0 and others are not compatible due to additional restrictions.

If you are unsure whether you can use a map or not, please do reach out and we will be able to help you. The following websites are good examples of where you can source open access or public domain maps: 
* U.S. Geological Survey (USGS) - All maps are in the public domain. (<http://www.usgs.gov>) 
* PlaniGlobe - All maps are published under a Creative Commons license so please cite “PlaniGlobe, <http://www.planiglobe.com>, CC BY 2.0” in the image credit after the caption. (<http://www.planiglobe.com/?lang=enl>) 
* Natural Earth - All maps are public domain. (<http://www.naturalearthdata.com/about/terms-of-use/>)

5. In the online submission form, you indicated that your data is available only on request from a third party. Please note that your Data Availability Statement is currently missing [the name of the third party contact or institution / contact details for the third party, such as an email address or a link to where data requests can be made]. Please update your statement with the missing information.

- Amended- see lines 87-89.

6.We have amended your Competing Interest statement to comply with journal style. We kindly ask that you double check the statement and let us know if anything is incorrect.

- Correct

[Note: HTML markup is below. Please do not edit.]

Reviewers' comments:

Reviewer's Responses to Questions

5. Review Comments to the Author

Please use the space provided to explain your answers to the questions above. You may also include additional comments for the author, including concerns about dual publication, research ethics, or publication ethics. (Please upload your review as an attachment if it exceeds 20,000 characters)

Reviewer #1:

- Abstract: I think that the conclusion is too strong. A ecological study cannot demonstrate an hypothesis. Consider using “suggest” or “support”.

- Thank you for your comment. This has been amended, line 39

- As temperature is a major confounder, I wonder how the model including only temperature has improved when the number of COVID diagnoses were added to the model. Could authors describe? Maybe using LR test.

- Amended, line 175-179, 198-200.

- I suggest that the information in this paper could be relevant to the discussion and support the use of ecological methods. COVID toes were commonly associated with positive results in the family members, not the patient. doi: 10.1016/j.annder.2020.11.005.

- Thank you for the helpful reference. This has been included in lines 267-273.

Reviewer #2:

I have one major concern that should be addressed:

- The authors conclude that they demonstrate a method that could support tracking of COVID-19 viral spread in the community. I strongly disagree with this finding. The study included a subset of 3,687 confirmed cases of COVID-19 at a time where the number of confirmed cases in the population under study was close to 40,000. I believe this study's findings are strongly biased by increased likelihood of patients presenting to a GP if they had COVID-19 and then also subsequently developed chilblains. The study is still publishable, but with the removal of the conclusion for the method having potential as a surrogate measure for the spread of COVID-19 infections. I suggest the conclusion should be more focused on increasing clinician awareness of the potential for chilblains in their COVID-19 patients - not anything to do with surveillance for COVID-19.

- Thank you for your comments. We agree that the power of the study may not be sufficient to claim that chilblains could be used as a surrogate public health measure of COVID-19 but aimed to demonstrate the potential utility of the ecological technique we used. We have now amended as per your suggestion. See lines 43-45, 305-308.

Other minor comments are:
- Lines 145-146 - sentence starting with We obtained ... does not make sense - use of BOM

- Amended, line 154-155.

- The increased RR for 0-16 year olds is likely associated with the increased likelihood of parents to take their children to the GP with the combined conditions. This should be made clear in the paper and references included to show that this is a known phenomenon.

- Our data demonstrates the inverse, with a proportional representation of children in our dataset compared to the underlying population. Additionally Australian studies have shown a reduction in health service utilisation throughout the pandemic. This has been outlined in lines 250-254.

- Please label Table 1 more appropriately, footnote any abbreviations, and include numbers in the Table.

- Amended, see table 1 and footnotes on page 10
